# Complete Genome Sequences of Two Distinct Strains of Serratia marcescens Isolated from Contaminated Platelet Concentrates from Canadian Donors

**DOI:** 10.1128/MRA.00829-20

**Published:** 2020-10-08

**Authors:** Alexander Diamandas, Roel Mikhail Razon, Ellen Sykes, Ayush Kumar, Sandra Ramirez-Arcos, Ann Karen C. Brassinga

**Affiliations:** a Department of Microbiology, University of Manitoba, Winnipeg, Canada; b Canadian Blood Services, Ottawa, Canada; c Department of Biochemistry, Microbiology and Immunology, University of Ottawa, Ottawa, Canada; University of Maryland School of Medicine

## Abstract

In this report, we present the genome sequences of two Serratia marcescens strains isolated as contaminants from platelet concentrates by Canadian Blood Services and designated CBS2010/11 (CBS11) and CBS2010/12 (CBS12). Genomic sequence analyses showed that CBS11 has one chromosome and one plasmid (pAM01), whereas CBS12 has no plasmids.

## ANNOUNCEMENT

Serratia marcescens is a common bacterial contaminant found in platelet concentrates (PCs) and has been associated with adverse transfusion reactions in recipient patients (1). Sources of bacterial contamination in PCs may be from the blood donor, from either the collection of skin flora at the venepuncture site or transient bacteremia in the blood, or incurred during PC processing, of which the common source is skin flora (2). The physicochemical conditions (neutral pH, high glucose content, and 22°C with agitation) of stored PCs promote the proliferation of bacterial contaminants (1). S. marcescens is particularly adept as an elusive pathogen due to its ability to survive skin disinfection and thrive in PCs by forming biofilms on the interior surface of the plastic storage bag (1, 3). The sequences presented here are of two unrelated strains of S. marcescens detected by routine BacT/Alert screening of PCs (1).

The genomes of strains CBS2010/11 (CBS11) and CBS2010/12 (CBS12) were sequenced and assembled using Pacific Biosciences (PacBio) technology (Menlo Park, CA, USA). Briefly, the strains were grown overnight in Luria-Bertani (LB) broth (Difco), and genomic DNA was extracted using the Purelink genomic DNA kit (ThermoFisher) in accordance with the manufacturer’s instructions. The purity and quantity of DNA were evaluated by a Nanodrop instrument and by an Invitrogen Qubit 4 fluorimeter, respectively, prior to submission to the Génome Québec facility. A total of 5 μg of high-molecular-weight genomic DNA (final volume of 80 μl) was sheared using g-TUBEs (Covaris, Inc.) at 4,000 rpm for 60 seconds on each side, on the centrifuge 5424 (Eppendorf). The DNA libraries were prepared in accordance with the PacBio 20-kb template preparation kit using the BluePippin size selection system protocol that includes the use of the SMRTbell template prep kit 1.0 reagents for DNA damage and end repair and ligation steps. The DNA library was size selected on a BluePippin system (Sage Science, Inc.) using a cutoff range of 12 kb to 50 kb. The sequencing primer was annealed at a final concentration of 0.8333 nM, and the P6 v2 polymerase was bound at 0.500 nM. The libraries were sequenced on a PacBio RS II instrument at a loading concentration (on plate) of 140 pM using the MagBead OneCellPerWell loading protocol, DNA sequencing kit 4.0 v2, and single-molecule real-time (SMRT) cells v3 with 240-minute movie collection. The combined data consisted of 110,975 reads with an *N*_50_ size of 13.4 kb for CBS11 and 111,386 reads with an *N*_50_ size of 13.5 kb for CBS12. The continuous long reads (CLR) were assembled *de novo* with the PacBio SMRT analysis software (7.0.0.63985) for the CBS11 genome and SMRT analysis software (6.0.0.47841) for CBS12, using the HGAP4 protocol (SMRTLink v6.0.0). A subset of reads were subjected to BLAST analyses to ensure quality control and authenticity of the genome assembly. Contig end trimming and circularization with Circlator (v1.5.5) were performed, followed by polishing with Quiver by the Canadian Center for Computational Genomics (C3G). Once polished, sequences were annotated with Prokka (v1.12) (4) referencing a genus-specific genome (NCBI number ASM78391v2) produced by the University of Maryland School of Medicine and Institute for Genome Science (IGS). In all tool applications, default parameters were used except where otherwise noted. The CBS11 genome sequence resolved into 2 contigs, namely, a 5,352,713-bp contig and a 139,857-bp contig, representing a chromosome and a plasmid, respectively, with a G+C content of 59.39%, that combined included 4,950 genes, 22 rRNAs, 101 tRNAs, and 1 noncoding RNA. The CBS12 genome sequence resolved into 1 5,642,069-bp contig ascribed to the sole chromosome containing 5,204 genes, 22 rRNAs, 93 tRNAs, and 1 noncoding RNA.

Genome sequence analyses of CBS11 and CBS12 indicated that these two isolates are not clones and therefore are unique strains. Furthermore, a majority of the pAM01 plasmid sequence shares high identity (>90%) with S. marcescens plasmid PWNM146 (GenBank accession number LT575492) (5). Further genomic analyses are ongoing to understand the ability of these two S. marcescens strains to survive and thrive in PCs.

### Data availability.

This genome project has been deposited in GenBank under accession numbers CP053927 (chromosome of CBS11), CP053926 (plasmid pAM01), and CP053925 (chromosome of CBS12). Accession numbers for raw reads for the CBS11 chromosome and plasmid pAM01 and for the CBS12 chromosome are PRJNA634532 and PRJNA634544, respectively.
